# Critical Prognostic Parameters in the Anatomic Pathology Reporting of Differentiated Follicular Cell-Derived Thyroid Carcinoma

**DOI:** 10.3390/cancers11081100

**Published:** 2019-08-02

**Authors:** Bin Xu, Ronald Ghossein

**Affiliations:** Department of Pathology, Memorial Sloan-Kettering Cancer Center, New York, NY 10021, USA

**Keywords:** extrathyroidal extension, lymphovascular invasion, capsular invasion, AJCC staging

## Abstract

In the past decades, pathology reporting on thyroid carcinoma has evolved from a narrative approach to structured synoptic reports. Many histologic variables are present in the current synoptic reports that are crucial elements for initial risk stratification and clinical management. In this review, we compare and summarize the key prognostic pathologic characteristics utilized by the most influential clinical and pathologic guidelines from the American Thyroid Association (ATA), the National Comprehensive Cancer Network (NCCN), the current World Health Organization (WHO) classification of endocrine tumors (fourth edition), the current American Joint Committee on Cancer (AJCC) staging system (eighth edition), the College of American Pathologists (CAP) protocol, and the International Collaboration on Cancer Reporting (ICCR) dataset. The aim is to provide a comprehensive review focused on the definitions and prognostic impacts of these crucial pathologic parameters.

## 1. Introduction

In the modern era, a detailed cancer pathology report beyond a simple diagnosis and TNM (tumor, node, metastasis) staging provides crucial information for managing patients. The reporting of thyroid cancer is no exception to this. Multiple prognostically important histopathologic features have been adapted into standardized pathology reports over the years. These microscopic parameters (e.g., tumor subtyping, the existence and extent of capsular invasion and vascular invasion, extrathyroidal extension (ETE), margin status, number of lymph nodes involved by metastasis, size of metastasis) are prognostically relevant and have been used in clinical management for risk stratification to determine the most appropriate treatment approach. In this article, we reviewed and summarized crucial parameters in thyroid carcinoma reporting, focusing primarily on the major guidelines, including the American Thyroid Association (ATA) management guidelines published in 2015 [1]; the National Comprehensive Cancer Network (NCCN) clinical practice guidelines for thyroid carcinoma (version 1.2019) [2]; the World Health Organization (WHO) classification of tumor of endocrine organs (fourth edition) issued in 2017 [3]; the American Joint Committee on Cancer (AJCC) cancer staging manual (eighth edition) that appeared in 2017 [4]; the College of American Pathologists (CAP) protocol for the examination of specimens from patients with carcinomas of the thyroid gland (updated in February 2019) [5]; and the International Collaboration on Cancer Reporting (ICCR) thyroid dataset (2019) [6]. The aim of this review is to provide practicing thyroid pathologists a concise comparative overview of the different guidelines, focusing on those the prognostic factors in differentiated follicular cell-derived thyroid carcinoma that may influence clinical management.

## 2. Key Pathologic Parameters Currently Used in Thyroid Carcinoma Clinical Guidelines 

In patients with differentiated follicular cell-derived thyroid carcinoma, post-operative risk stratification and the decision to perform completion thyroidectomy and delivery of radioactive iodine therapy relies heavily on a detailed surgical pathology report [7]. Pathologic factors that are included in clinical management guidelines include: (1) tumor type and variants of papillary thyroid carcinoma (PTC); (2) the presence of lymphatic invasion [2] or vascular invasion [1] in PTC; (3) the extent of vascular invasion in follicular thyroid carcinoma (FTC) and Hurthle cell carcinoma (HCC); (4) gross and microscopic extrathyroidal extension into perithyroidal soft tissue or other loco-regional structures; (5) number of lymph nodes with metastases; and (6) size of the lymph node with metastasis; (7) margin status or gross residual disease; and (8) size of the primary tumor [1,2]. 

More importantly, it is crucial for pathologists to understand which tumor characteristics constitute intermediate- and high-risk lesions, as they may drive clinical decision-making for additional post-operative treatment. Based on the ATA [1] and NCCN [2] guidelines, the following pathologic parameters, if present, are considered as high-risk lesions: (1) the presence of poorly differentiated thyroid carcinoma (PDTC); (2) gross extrathyroidal extension (ETE); (3) gross residual disease/incomplete surgical resection; (4) large metastatic neck lymph nodes defined as ≥3 cm at the largest dimension; and (5) extensive vascular invasion defined as >4 foci in FTC and HCC. A high-dose radioactive iodine (RAI) ablation regimen is recommended for high-risk lesions, especially in patients with gross residual diseases. 

Intermediate-risk lesions include: (1) aggressive PTC variants (e.g., tall cell, columnar cell, and hobnail variants); (2) microscopic but not gross extrathyroidal extensions; (3) PTC with lymphatic or vascular invasion; (4) lymph node metastases in more than five lymph nodes, with the largest metastatic node measuring <3 cm; and (5) a primary tumor size of >4 cm. For patients with intermediate-risk lesions, post-operative RAI, in particular the low-dose 30 mCi regimen, can be considered and is selectively recommended. 

## 3. Tumor Types and Variants Associated with Aggressive Clinical Behaviors

**Figure 1:**
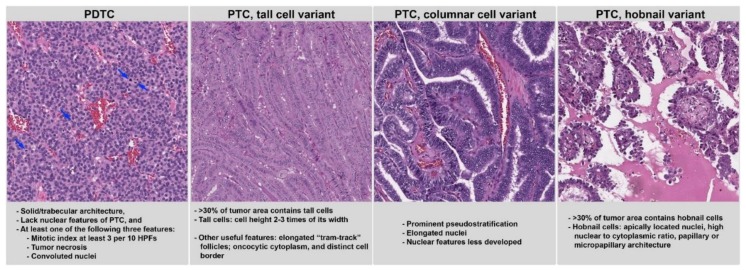
Histologic features and diagnostic criteria of aggressive types and variants of differentiated thyroid carcinoma, including poorly differentiated carcinoma (PDTC), papillary thyroid carcinoma (PTC) tall cell variant, columnar cell variant, and hobnail variant. The diagnostic features of each tumor are listed in the textbox below. HPFs: high power fields. Blue arrows: mitoses. Magnification: 100×.

The fourth edition of the WHO classifications recognizes four types of differentiated follicular cell-derived carcinomas: PTC, FTC, HCC, and PDTC [3]. Among them, PDTC is associated with the worst prognosis, with a five-year overall survival of 60–70% [8,9,10]. The current WHO classification defines PDTC using the criteria of the Turin proposal published in 2006 (Figure 1) [9]. In brief, a tumor is considered as PDTC when all of the following criteria are met: (1) solid/trabecular architecture; (2) absence of the nuclear features of PTC; and (3) tumor necrosis, a mitotic index ≥3 per 10 high power fields (HPFs), and/or convoluted nuclei. Additionally, Hitzik et al. have shown that high mitotic count (defined as ≥5/10 HPFs) and/or tumor necrosis is associated with an adverse outcome regardless of architectural pattern or nuclear features [10]. Tumors with a high mitotic count and/or tumor necrosis that retaining either nuclear features of PTC or non-solid architecture are referred as high-grade PTC or FTC by those using the Turin proposal [11], but we label them in our institution as PDTC (8). This is based on multivariate analysis showing that differentiated thyroid carcinomas with high mitotic activity and/or tumor necrosis behave similarly (overall in an aggressive fashion) regardless of their architectural and cytologic patterns [10]. Whatever the nomenclature used, given the associated adverse outcomes in tumors with mitotic activity and/or necrosis, it is prudent for pathologists to search for hotspot areas with increased mitotic activity or tumor necrosis in any thyroid carcinoma. With that in mind, the mitotic index (reported as the number of mitotic figures per 2 square mm) and tumor necrosis became the two mandatory reporting elements in the ICCR thyroid synoptic report [6].

There are more than 10 variants of PTC in the most recent WHO classification [3]. All major guidelines [1,2,3,4,5] recognized the tall cell, columnar cell, and hobnail variants as the more aggressive subtypes based on current evidence [12,13,14,15,16,17,18]. In the WHO classification, the diagnosis of tall cell and hobnail variants requires ≥30% of the total tumor to show typical features of tall cell or hobnail cytology [3]. There is no proposed percentage cutoff for columnar cell variants [3]. The diagnostic features of these three variants are highlighted in Figure 1. In brief, tall cell is defined as a cell whose height is two to three times its width. Hobnail cells typically exhibit apically located protruding nuclei with high nuclear/cytoplasmic ratios arranged as papillae and micropapillae. Columnar cell variants show prominent pseudostratification of cigar-shaped elongated nuclei with less evident PTC nuclear features.

## 4. Capsular Invasion 

**Figure 2:**
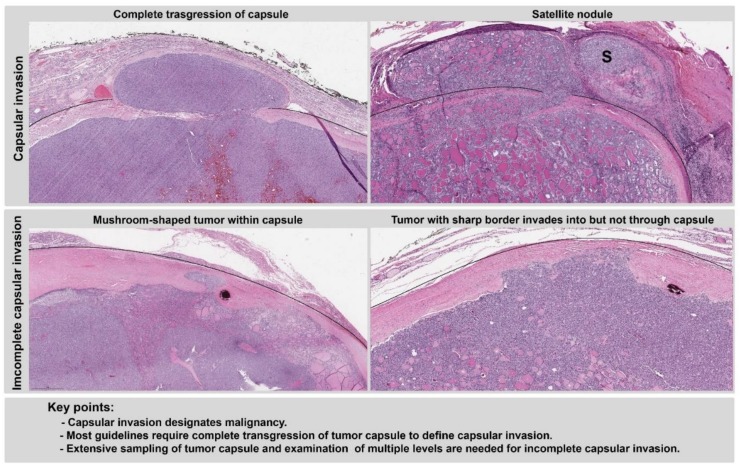
Capsular invasion. Upper panels: capsular invasion is defined as complete transgression of the capsule or satellite nodule(s) lying outside of the tumor capsule. Lower panels: incomplete capsular invasion is defined as incomplete penetration of the tumor capsule. Black lines outline the outer contour of tumor capsule. S: satellite nodule. Magnification: 40×.

The key points and representative samples of capsular invasion are listed in Figure 2. The WHO classification [3], CAP checklist [5], and ICCR dataset [6] recognize complete transgression of a tumor capsule as capsular invasion, with or without neocapsule formation. Satellite nodules with similar architectural and cytologic features lying outside the tumor capsule are also considered capsular invasion by the AFIP (Armed Forces Institute of Pathology) fascicles, CAP checklist, and ICCR dataset [5,6,19]. Capsular invasion is not a parameter included in the clinical management guidelines (ATA [1] and NCCN [2]) for risk stratification, as follicular carcinoma with capsular invasion only is defined as minimally invasive follicular carcinoma in the most recent WHO classification, has an overall excellent prognosis and a metastatic risk close to zero [20,21,22,23,24,25]. However, the existence of invasion, including capsular invasion, separates malignant thyroid carcinoma (FTC, HCC, or the encapsulated follicular variant of PTC) from its non-malignant counterparts (follicular adenoma, Hurthle cell adenoma, or noninvasive follicular thyroid neoplasm with papillary-like nuclear features (NIFTP)) [26]. 

The approach to addressing tumors with incomplete or questionable capsular invasion differs among guidelines, although all guidelines recommended additional or complete sampling of the capsule and examination of additional histologic levels. In the CAP checklist [5], it is noted that some “authorities do not require complete transgression of the capsule” to define capsular invasion, and incomplete transgression “may be accepted to some pathologists as presenting capsular invasion”. In the WHO classification [3], a new category of tumors named “well-differentiated tumor of uncertain malignant potential” is included to describe tumors with questionable invasion—capsular or vascular—after thorough histologic examination. However, such designation has not yet gained popularity in North America. In the ICCR dataset [6], foci that remain questionable for capsular invasion despite enhanced histopathologic examination are reported as uncertain capsular invasion.

## 5. Lymphovascular Invasion (LVI)

In thyroid carcinoma, the type of carcinoma usually designates the route of spread [3]. While follicular-patterned lesions (HCC, FTC, and encapsulated follicular variant of PTC) usually metastasize distantly and bypass lymph nodes, PTC, in particular classic and tall cell variants, tends to spread to lymph nodes. Therefore, the most common type of LVI in a follicular-patterned lesion is vascular invasion, and in a classic or tall cell PTC is lymphatic invasion. 

The last three editions of the CAP checklist [5] have separated LVI into lymphatic invasion and vascular invasion. Such an approach has drawbacks, however. First, there is no clear-cut histologic definition of lymphatic invasion in thyroid. Secondly, there are no reliable features to separate lymphatic from vascular invasion based on histology alone. The CAP checklist has proposed using the caliber of the vessel, the presence of red blood cells, and D2-40/CD31 immunostain to distinguish vascular invasion from lymphatic invasion. However, there are large-caliber lymphatic vessels with muscular walls, and small-caliber capillaries are indistinguishable from small-caliber lymphatic channels. Although red blood cells should only exist in vascular channel in theory, it is not uncommon to have a capillary devoid of red blood cells, and a lymphatic channel containing carry-over red blood cells on hematoxylin and eosin (H&E) slides (especially in a vascular-rich organ such as thyroid). Distinguishing vascular invasion from lymphatic invasion by immunohistochemistry studies alone is not always possible, in our experience. Lately, this separation has spread into clinical management guidelines and created significant confusion. The NCCN guidelines, for example, have included a parameter named “degree of lymphatic invasion” in their management guidelines for FTC and HCC [2], whereas the ATA guidelines have used a single term, “vascular invasion”, throughout the publication for PTC, FTC, and HCC [1]. Recognizing the limitation of separating lymphatic from vascular invasion based on histologic examination alone, the upcoming ICCR dataset has once again merged lymphatic invasion and vascular invasion back into the single term “lymphovascular invasion” [6]. 

As multiple studies have shown that the existence and/or extent of vascular invasion predicts the risk of distant metastasis and clinical outcome in FTC and HCC [21,22,27,28,29,30], it becomes an important pathologic parameter that has been incorporated into clinical management guidelines [1,2]. Additionally, all pathology guidelines advocate the separation of FTC and HCC with LVI from those with capsular invasion alone as angioinvasive and minimally invasive FTC/HCC, respectively, in order to highlight the more aggressive nature of angioinvasive tumors [3,5,6]. 

**Figure 3:**
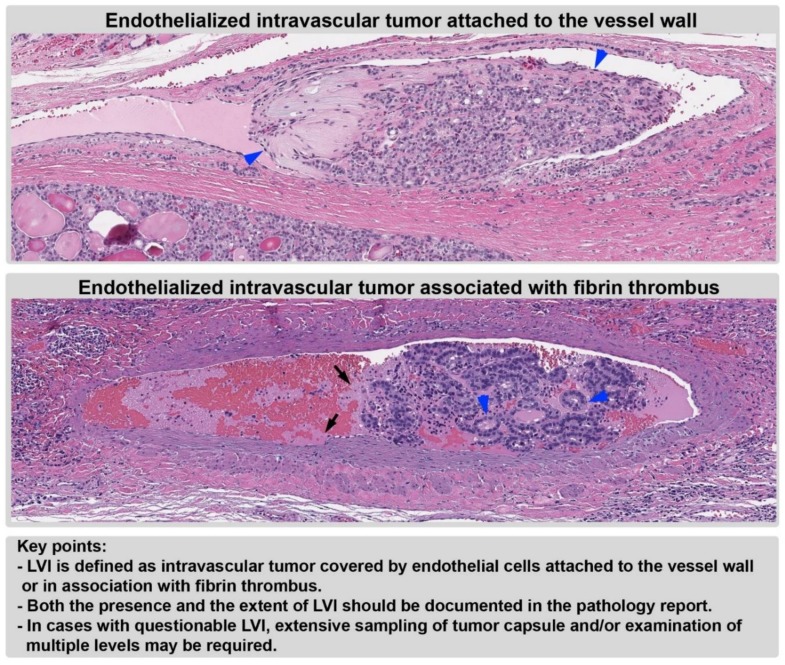
Lymphovascular invasion (LVI). LVI is defined as an intravascular tumor attached to the vessel wall or associated with fibrin thrombi. Blue arrowheads: endothelial cells. Black arrows: fibrin thrombus. Magnification: 200×.

However, the very definition of LVI in thyroid carcinomas has been surrounded by controversies. Traditionally, LVI is defined as an intravascular tumor attached to the vessel wall and covered by endothelial cells, as defined in the AFIP fascicle (Figure 3) [19]. This has been recently challenged by Mete and Asa [31] who showed that manipulation of the thyroid gland can result in the protrusion of a tumor covered by endothelial cells into the vascular lumen. Therefore, the authors have proposed to define vascular invasion only when the tumor embolus is associated with fibrin thrombus. This more stringent criteria result in three changes: (1) a drastic decrease in LVI frequency from 7–62% [22,30,32,33,34] to 3%; (2) a high risk of distant metastasis (35%) in tumors with LVI, even when only one focus is present: and (3) a diminished impact of the extent of LVI in prognosis. Given the 35% risk of distant metastasis, LVI in association with fibrin thrombus is clearly highly significant clinically. However, it may underestimate the frequency of actual LVI and its associated risk of distant metastasis, as no studies to date have shown that LVI without associated fibrin carries no risk of distant metastasis. Major guidelines have dealt with this issue differently; while the WHO classification [3] and ICCR dataset [6] define LVI as an intravascular tumor attached to the vessel wall, covered by endothelium or associated with fibrin thrombus, the CAP protocol [5] has endorsed the paper from Mete and Asa (i.e., only those intravascular tumor cells with associated fibrin thrombus are considered as convincing LVI, whereas an endothelialized tumor deposit without obvious thrombus “would not count as significant VI” and “may still be considered as a judgement call”).

In regard to extent of vascular invasion, it remains a crucial element for clinical decision making in the ATA and NCCN guidelines [1,2]. At present, FTC and HCC with focal (less than four foci) vascular invasion are considered low risk by the ATA, and thus do not require RAI ablation, whereas extensive vascular invasion is considered a high-risk lesion by the ATA, for whom RAI is recommended [1]. Similarly, in the NCCN guidelines, RAI is recommended for FTC/HCC with extensive vascular invasion, whereas it is only selectively recommended for those with minor vascular invasion (one to four foci) [2]. The notion of treating a tumor with focal LVI more conservatively is based on the observation that focal LVI is associated with a low risk of recurrence (0–5%) and an overall similar prognosis to tumors without VI [21,22,27,28,29]. Extensive vascular invasion on the other hand is associated with a 42% risk of distant metastasis, and is commonly seen in patients with recurrence and/or disease-specific death [22,28,35,36]. In view of its use in all clinical guidelines, reporting the extent of LVI in thyroid carcinomas, especially follicular and Hurthle cell carcinoma, is recommended.

## 6. Extrathyroidal Extension (ETE)

**Figure 4:**
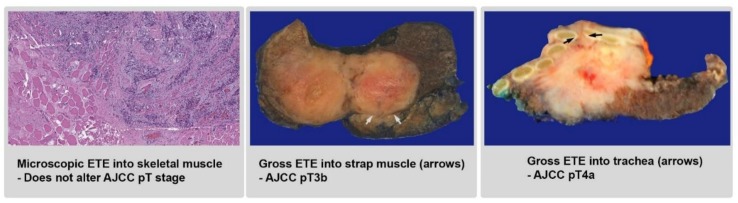
Extrathyroidal extension (ETE). Microscopic ETE does not affect American Joint Committee on Cancer (AJCC) pT staging, whereas gross ETE into strap muscle or adjacent organs is considered AJCC pT3b and pT4a, respectively. Magnification of microscopic picture: 40×.

One significant change in the AJCC eighth edition staging for thyroid carcinoma is that microscopic extrathyroidal extension (ETE), defined as tumor extension beyond the thyroid capsule into either perithyroidal soft tissue or skeletal muscle, is no longer a factor in tumor staging and is no longer considered as pT3 disease (Figure 4) [4]. This change is based on the observation that microscopic ETE is not an independent prognostic factor for disease-free or disease-specific survival, and is associated with a low risk (3–9%) of recurrence [36,37,38,39,40,41,42,43,44,45]. In contrast, gross ETE (defined as extension into strap muscle, subcutaneous tissue, adjacent organs (e.g., larynx, esophagus, or trachea), or the recurrent laryngeal nerve) observed radiologically, at the time of operation, or during macroscopic examination, correlates with a high recurrence risk of 23–40% [36,37,38,40,41,42,46]. Gross ETE is also considered a high-risk parameter in the ATA guidelines [1]. The determination of gross ETE into strap muscle (AJCC pT3b) or into adjacent organs (AJCC pT4a) relies heavily on clinical findings, especially pre-operative high-resolution imaging and intra-operative findings. As gross ETE becomes a crucial parameter in determining pathologic staging, the upcoming ICCR dataset [6] mandates the documentation of the presence and site of gross ETE with a caveat that such information is not always available to pathologists and often depends on the operating surgeon. 

Although microscopic ETE is no longer a factor in AJCC staging, it is still included in the clinical guidelines. In the ATA guidelines, microscopic ETE is considered a parameter for intermediate risk, a category in which RAI ablation may be considered in select patients [1]. In the NCCN guidelines, a low-dose regimen of 30 mCi RAI may be administered to patients with tumors exhibiting microscopic ETE [2]. Unfortunately, the diagnosis of microscopic ETE can be challenging. Histologically, the thyroid gland does not have a complete capsule. At the periphery of the thyroid, follicles are often seen intermingled with adipose tissue. At the level of isthmus, thyroid tissue can be seen admixed with skeletal muscle [47]. Therefore, it is not surprising that the definition of microscopic ETE lacks universal agreement. A recent study involving 11 expert endocrine pathologists demonstrated the difficulty in arriving at a consensus. Among the 11 pathologists involved, the interobserver agreement for microscopic ETE was poor, with a kappa value of 0.14 [48]. The only universally accepted criterion for microscopic ETE was invasion into perithyroidal skeletal muscle outside of the isthmus area, whereas other criteria (e.g., involvement of the perithyroidal nerve, thick-wall vessels, fibroadipose tissue, or skeletal muscle at the isthmus) were only agreed upon by the majority, not by all expert pathologists [48]. Such differences of diagnostic criteria are also reflected in the pathology guidelines—the CAP protocol only recognizes invasion into strap muscle as ETE [5], whereas the ICCR dataset considers both extension into perithyroidal soft tissue and into skeletal muscle as microscopic ETE [6]. 

## 7. Margins of Resection

There is a general consensus among thyroid pathologists that a positive resection margin is defined by thyroid carcinoma cells present on the inked tissue margin [49,50,51,52]. Since several publications have demonstrated that a microscopic positive margin is not an independent predictor of outcome [49,50,51,52], macroscopic tumor resection leaves the patient in the low risk category in the current ATA guidelines, while incomplete tumor resection (i.e., a grossly positive margin) is a defining feature of high-risk tumors, prompting more aggressive therapy [1]. The assessment of the clinically relevant margins is therefore dependent on both the pathologist and the operating surgeon. In regard to close margins (i.e., tumors close to the inked margin), there is no data on its clinical significance, to our knowledge. Consequently, it is not mandatory to measure the distance between tumor and margins [5,6].

## 8. Regional Lymph Node Metastasis

Nodal metastasis is a common finding in PTC, affecting up to 80% of the patients [53]. It is a component of AJCC TNM staging. N1 disease can be further divided into pN1a (central compartment or upper mediastinal lymph nodes) and pN1b (lateral compartment or retropharyngeal lymph nodes) based on the location of positive lymph nodes [4]. In addition to the AJCC nodal staging, additional features (e.g., the number of involved lymph nodes and the size of the largest metastatic node), have been included in the ATA and NCCN guidelines as elements for risk stratification [1,2]. A recent meta-analysis by Randolph et al. has shown that microscopic pathologic N1 disease, defined as fewer than five positive lymph nodes and none >1 cm in size, does not significantly impact survival of patients with PTC [53]. Therefore, small-volume nodal disease (defined by the ATA as fewer than five positive lymph nodes with metastatic foci <2 mm and by the NCCN as fewer than three to five positive lymph nodes with a 2–5 mm metastatic focus) is considered low risk, which does not mandate post-operative RAI ablation. On the other hand, a large metastatic node (≥3 cm) is classified as high risk by the ATA. Both CAP and ICCR guidelines require detailed reporting of nodal status, including location of the involved lymph node(s), number of positive lymph nodes, and sizes of the largest metastatic nodes and largest metastatic focus [5,6]. Table 1 provides a comparison of pathologic parameters among various guidelines.

## 9. Conclusions

A detailed pathology report is now needed to manage thyroid carcinoma patients post-operatively. The key pathologic features that guide clinical decision making for follicular cell-derived carcinoma include histologic type, histologic variant, capsular invasion, vascular invasion, extrathyroidal extension, and details on regional lymph node metastasis. Some of these features, such as vascular invasion or extra-thyroid extension, require considerable expertise and are best analyzed by a pathologist with special knowledge of thyroid neoplasia. The ensuing risk stratification accuracy will help many patients get the needed treatment, while sparing many unnecessary aggressive therapies and side effects.

## Figures and Tables

**Table 1:** Comparison of the key pathologic prognostic factors among different guidelines.

Pathologic Parameters	WHO/AJCC	ICCR	CAP
Aggressive types of differentiated thyroid carcinoma	Poorly differentiated thyroid carcinoma Papillary thyroid carcinoma, tall cell variant, columnar cell variant, and hobnail variant		
Definition of capsular invasion	Complete penetration of tumor capsule *		
Lymphovascular invasion (LVI), definition	Intravascular tumor attached to vessel wall, covered by endothelium or associated with fibrin	Intravascular tumor attached to vessel wall, covered by endothelium or associated with fibrin	Intravascular tumor should be accompanied by fibrin thrombus. Endothelial-covered tumor not considered LVI
Separation of lymphatic and blood vessel invasion	No clear position	Does not mandate separation of lymphatic and blood vessel invasion	Mandate separation of lymphatic invasion and blood vessel invasion
Extent of lymphovascular invasion (No. of foci)	Tumors with limited invasion (<4 foci) have a better prognosis than those with extensive invasion; no clear position in regard to reporting	Tumors with limited invasion (<4 foci) have a better prognosis than those with extensive invasion; mandatory reporting Focal: <4 foci Extensive: ≥4 foci	Prognostic value of extent of invasion controversial; optional reporting Focal: <4 foci Extensive: ≥4 foci
Extrathyroidal extension (ETE)	Microscopic ETE does not alter the T stage Gross ETE into strap muscle (pT3b) requires reviews of macroscopic, intraoperative, and/or radiologic findings		
Mitosis/tumor necrosis	Crucial criterion in definition of poorly differentiated thyroid carcinoma; no clear position in regard to reporting	Crucial criterion in definition of poorly differentiated thyroid carcinoma; mandatory reporting	Crucial criterion in definition of poorly differentiated thyroid carcinoma; optional reporting
Margin status	Issue not addressed	Positive (tumor at resection edge); mandatory reporting of positive/negative status; optional reporting of site/margin clearance	Positive (tumor at resection edge); mandatory reporting of positive/negative status; optional reporting of site/margin clearance

* All guidelines agree with this definition. The approach for incomplete capsular invasion (CI) varies across different guidelines. AJCC: American Joint Committee on Cancer; WHO: World Health Organization, ICCR: International Collaboration on Cancer Reporting; CAP: College of American Pathologists.
